# Exposure to air pollutants and subclinical carotid atherosclerosis measured by magnetic resonance imaging: A cross-sectional analysis

**DOI:** 10.1371/journal.pone.0309912

**Published:** 2024-10-31

**Authors:** Sandi M. Azab, Dany Doiron, Karleen M. Schulze, Jeffrey R. Brook, Michael Brauer, Eric E. Smith, Alan R. Moody, Dipika Desai, Matthias G. Friedrich, Shrikant I. Bangdiwala, Dena Zeraatkar, Douglas Lee, Trevor J. B. Dummer, Paul Poirier, Jean-Claude Tardif, Koon K. Teo, Scott Lear, Salim Yusuf, Sonia S. Anand, Russell J. de Souza, for the Canadian Alliance of Healthy Hearts and Minds (CAHHM) Study Investigators

**Affiliations:** 1 Department of Health Research Methods, Evidence, and Impact, McMaster University, Hamilton, Ontario, Canada; 2 Department of Medicine, McMaster University, Hamilton, Ontario, Canada; 3 Research Institute of McGill University Health Centre, Montreal, Canada; 4 Population Health Research Institute, Hamilton, Ontario, Canada; 5 Occupational and Environmental Health Division, Dalla Lana School of Public Health, University of Toronto, Toronto, Ontario, Canada; 6 School of Population and Public Health, The University of British Columbia, Vancouver, British Columbia, Canada; 7 Department of Clinical Neurosciences, University of Calgary, Calgary, Alberta; 8 Department of Medical Imaging, Sunnybrook Health Sciences Centre, University of Toronto, Toronto, Ontario, Canada; 9 Department of Medicine and Diagnostic Radiology, McGill University, Montreal, Quebec, Canada; 10 Department of Biomedical Informatics, Harvard Medical School, Boston, Massachusetts, United States of America; 11 Programming and Biostatistics, Institute for Clinical Evaluative Sciences, Toronto, Ontario, Canada; 12 Faculté de Pharmacie, Institut Universitaire de Cardiologie et de Pneumologie de Québec, Quebec City, Quebec, Canada; 13 Montreal Heart Institute, Université de Montréal, Montreal, Quebec, Canada; 14 Faculty of Health Sciences, Simon Fraser University, Burnaby, British Columbia, Canada; University of Health Sciences Lahore, PAKISTAN

## Abstract

**Objectives:**

Long-term exposure to air pollution has been associated with higher risk of cardiovascular mortality. Less is known about the association of air pollution with initial development of cardiovascular disease. Herein, the association between low-level exposure to air pollutants and subclinical carotid atherosclerosis in adults without known clinical cardiovascular disease was investigated.

**Design:**

Cross-sectional analysis within a prospective cohort study.

**Setting:**

The Canadian Alliance for Healthy Hearts and Minds Cohort Study; a pan-Canadian cohort of cohorts.

**Participants:**

Canadian adults (n = 6645) recruited between 2014–2018 from the provinces of British Columbia, Alberta, Ontario, Quebec, and Nova Scotia, were studied, for whom averages of exposures to nitrogen dioxide (NO_2_), ozone (O_3_), and fine particulate matter (PM_2.5_) were estimated for the years 2008–2012.

**Main outcome measure:**

Carotid vessel wall volume (CWV) measured by magnetic resonance imaging (MRI).

**Results:**

In adjusted linear mixed models, PM_2.5_ was not consistently associated with CWV (per 5 μg/m^3^ PM_2.5_; adjusted estimate = -8.4 mm^3^; 95% Confidence Intervals (CI) -23.3 to 6.48; p = 0.27). A 5 ppb higher NO_2_ concentration was associated with 11.8 mm^3^ lower CWV (95% CI -16.2 to -7.31; p<0.0001). A 3 ppb increase in O_3_ was associated with 9.34 mm^3^ higher CWV (95% CI 4.75 to 13.92; p<0.0001). However, the coarse/insufficient O_3_ resolution (10 km) is a limitation.

**Conclusions:**

In a cohort of healthy Canadian adults there was no consistent association between PM_2.5_ or NO_2_ and increased CWV as a measure of subclinical atherosclerosis by MRI. The reasons for these inconsistent associations warrant further study.

## Introduction

Cardiovascular disease (CVD) is a leading cause of mortality in Canada and worldwide, and traditional risk factors include smoking, obesity, diabetes, hypertension, and dyslipidemia [1]. Growing epidemiological evidence describes the adverse effects of air pollution on cardiovascular health [2–5], however the role of low levels of exposure to air pollution on early subclinical markers of cardiovascular dysfunction, *e*.*g*., atherosclerosis, is not well-characterized [6].

Major pollutants include particulate matter (PM) and gaseous air pollutants, such as nitrogen oxides (NO_x_) and ground level ozone (O_3_) [7]. Fine particulate matter (PM_2.5_) is fine inhalable particles ≤2.5 μm in aerodynamic diameter. Nitrogen dioxide (NO_2_) is mainly associated with road traffic and other forms of fossil combustion and is a precursor to O_3_, which is formed through chemical reactions between NOx and volatile organic compounds (VOC) in the presence of sunlight [8]. The 2022 special report by the Health Effects Institute (HEI) on long-term exposure to traffic-related air pollution (TRAP), which included 57 studies investigating cardiometabolic effects found that the confidence in the evidence for the association of air pollution with cardiovascular (circulatory and ischemic heart disease) mortality is high, but with cardiovascular morbidity is at best moderate to low [9, 10]. However, the methods of outcome assessment varied substantially and only half of the studies entered a meta-analysis of which one-third rated as high risk of bias for the confounder domain [9, 10].

Imaging of the carotid arteries is a non-invasive biomarker of subclinical atherosclerosis -the progressive buildup of plaques- for early prediction of IHD risk in healthy individuals without clinically significant CVD [11]. In the longitudinal Multiethnic Study of Atherosclerosis (MESA), PM_2.5_, NO_2_, and NO_X_ were not associated with carotid intima-media thickness (cIMT) change, while ambient O_3_ was associated with increased progression of cIMT [2, 8]. Magnetic resonance imaging (MRI) can accurately assess the presence of subclinical cerebrovascular atherosclerosis [11] by measuring carotid vessel wall volume (CWV) i.e. the entire thickness of the wall. MRI-determined CWV, compared to ultrasound-measured cIMT, includes the adventitia, which is the source of vasa vasorum that further proliferates with arterial wall thickening [12]. Thus, CWV is a more sensitive measure of early plaque development [12, 13] and more consistently associated with incident CVD than cIMT [14]. To date, the association of ambient air pollution and MRI-captured CWV has not been studied. In the Canadian Alliance for Healthy Hearts and Minds Cohort (CAHHM) [15] of generally healthy adults, we sought to characterize the associations between low levels of exposure to PM_2.5_, NO_2_, and O_3_, and MRI-measured carotid atherosclerosis as a major CVD pathway.

## Methods

### Study design and participants

The design and methods of the CAHHM prospective cohort study have been previously described [15]. Participants were recruited from January 1, 2014 to December 31, 2018 from the provinces of British Columbia, Alberta, Ontario, Quebec, and Nova Scotia in mostly urban locations [15]. Research ethics board approval was obtained from the Hamilton Integrated Research Ethics Board (HiREB # 13–255), and all participants provided written informed consent. All data were deidentified. The cohort includes 8258 adults from across Canada, of whom > 80% were participants in ongoing prospective cohort studies and assessed for CVD traditional risk factors. MRI scans of the brain, heart, carotid artery, and abdomen were performed at enrollment, and 7973 participants completed a standard carotid MRI scan. Adults with known history of CVD (defined as a self-reported history of stroke, coronary artery disease, heart failure, or other heart disease), incomplete data on the non-lab based cardiovascular risk score, or incomplete air pollutants values were excluded for the presented analyses, leaving a final sample size of 6645 participants (S1 Fig).

### Assessment of air pollution exposure

The three major air pollutants of interest in this study were PM_2.5_, NO_2_, and O_3_. The development of these exposure datasets has been documented elsewhere [16–18] and they have been used in multiple Canadian epidemiological studies, including the recent Mortality-Air Pollution Associations in Low Exposure Environments (MAPLE) study [19]. Briefly, annual average exposures for the five years prior to the start of CAHHM recruitment (2008–2012), data distributed by the Canadian Urban Environmental Health Research Consortium (CANUE) [20] (www.canue.ca), were linked to CAHHM using the six-character residential postal code of participants at the time of recruitment. The average exposure over the five years prior to recruitment was chosen as it is considered to be representative of long-term air pollution exposure gradients and relevant for investigating subclinical CVD, which manifests over a long period of time [21].

Key emission sources for PM_2.5_ are industrial emissions, wildfire smoke, space heating, residential wood heating, cooking, agriculture, and vehicle traffic emissions. A significant fraction of PM_2.5_ is a result of atmospheric chemistry, forming from a range of gaseous precursors such as sulphur dioxide (SO_2_), NO_2_, ammonia (NH_4_), and volatile and semi volatile organic compounds. Yearly averages of PM_2.5_ concentrations prior to baseline assessment were estimated across a 1x1 kilometer grid covering North America using NASA MODIS, MISR, and SeaWIFS satellite instruments, with aerosol vertical profiles and scattering properties simulated by the GEOS-Chem chemical transport model [16]. To adjust for any residual bias in the satellite-derived PM_2.5_ estimates, a geographically weighted regression (GWR) incorporating ground-based observations was then applied [16]. Good agreement was found with cross-validated surface observations across North-America (R^2^ = 0.70). For most residential addresses, postal code areas were considerably smaller than 1x1 km so that the assigned PM_2.5_ concentration matches the 1x1 km grid square that the postal code is found within. Specifically, assigning PM_2.5_ to postal codes was performed using the single linkage approach where the PM_2.5_ grid square selected was the one closest to the x, y coordinate within a postal code polygon that best represents where the majority of the population lived.

NO_2_ is considered an indicator of TRAP, which is a complex mixture of gases and particles, including ultrafine particles (diameter ≤ 0.1 μm). Annual average NO_2_ concentrations in parts per billion (ppb) were estimated for each postal code location using a national land use regression (LUR) model for the year 2006 [17] at 100 m resolution and adjusted for prior and subsequent years using long-term air quality monitoring station data. The LUR NO_2_ model included road length, 2005–2011 satellite NO_2_ estimates, area of industrial land use within 2 km, and summer rainfall as predictors of regional NO_2_ variation [17]. Deterministic gradients were used to model local scale variation related to roads (i.e. traffic) [17]. The final NO_2_ model showed good performance, explaining 73% of the variation in measurements from national air pollution surveillance (NAPS) monitoring data with a root mean square error (RMSE) of 2.9 ppb.

O_3_ is a photochemically produced oxidant gas that results from the reaction between sunlight and NOx and VOCs emitted from various natural sources and human activities such as fossil fuel combustion and wood combustion. Annual mean concentrations of O_3_ exposure at 10–15 km resolution were estimated using the GEM-MACH (Global Environmental Multi-scale—Modelling Air Quality and Chemistry) air quality forecast model combined with observations from monitoring networks [18, 22].

### Subclinical MRI outcomes

Details of the CAHHM MRI protocol have been previously published [15]. The protocol used validated standard techniques to collect information on morphology, function and tissue characteristics. Briefly, participants underwent a short non-contrast enhanced scan using a 1.5 or 3.0 Tesla magnet. Each of the centres underwent a validated test scan for quality assurance. Carotid artery vessel wall volume (mm^3^) (left, right, and combined) within a 32-mm vessel length centred on each carotid bifurcation (to include distal common and proximal internal carotid arteries) was measured by subtracting lumen volume from total vessel volume. The lumen was defined semi-automatically from axial bright blood images of the time of flight sequence which were reconstructed at 2 mm intervals. The outer wall of the carotid artery was semi-automatically defined and adjusted as needed by expert readers. The area of the vessel wall in each image was estimated by subtracting the lumen area from the outer wall vessel area. Vessel wall volume per slice was calculated by multiplying by 2 mm per slice. Vessel wall volumes for right and left carotid arteries were estimated by integrating the volume for the total number of slices for each artery. We used the maximum of either the left or right CWV as the measure of atherosclerosis in this study.

### The INTERHEART risk score

The non-laboratory-based INTERHEART risk score is a validated tool developed to estimate a person’s myocardial infarction (MI) risk based on a compilation of risk factors [23]. These include age, sex, smoking, second-hand smoke exposure, diabetes, high blood pressure, and family history of MI, waist-to-hip ratio (WHR); home or work social stress, depression, simple dietary questions, and physical activity [1]. The score ranges from 0 to 48 and is categorized into low- (a score ≤ 9), moderate- (a score of 10 to 15), or high- (a score ≥ 16) risk categories and is significantly associated with diagnosed CVD, and also the presence of subclinical cerebrovascular disease without known clinical CVD [11, 23].

### Definitions

Individual socioeconomic status: Education was categorized as the highest level of education attainment (High school or less, College or Trade, or University Degree). Employment status was categorized as employed, retired or unemployed. An indicator variable was used to identify individuals who traveled outside of their community of residence for work.

Neighbourhood socioeconomic status: Area-based social deprivation index and material deprivation index from 2011 Canadian census data linked to participants six-character postal code through CANUE databases were used to represent the socioeconomic status of the local community/society to which participants belonged [24]. The indices are the first two components of a principal component analysis (PCA) of the following six variables: the proportion of persons without a high school diploma; the employment population ratio; the average personal income; the proportion of persons living alone; the proportion of individuals separated, divorced or widowed; and the proportion of single-parent families [24].

Neighbourhood walkability: Walkability measures the degree to which a neighbourhood supports walking and was included because, in a nationally representative Canadian study (n = 1.8 million participants) with a 15-year follow-up, neighbourhood walkability was associated with reduced risk of cardiovascular mortality (HR: 0.91 [0.88, 0.95]) [25]. Participants residential postal codes were linked to the Canadian Active Living Environments Index (Can-ALE) categorical variable that characterizes the favourability/friendliness of active living (i.e. walkability) potential of neighbourhoods in census metropolitan areas (CMA) on a scale from 1 (very low) to 5 (very high) based on intersection density, dwelling density, and points of interest measures available for the year 2016. An environment with a very high walkability tends to be densely populated and has very connected street patterns and a variety of walking destinations. Consequently, such environments are more urbanized and tend to experience higher NO_2_ levels. More information on Can-ALE is available at: http://canue.ca/wp-content/uploads/2018/03/CanALE_UserGuide.pdf.

### Statistical analysis

The distribution of continuous variables is presented as means with standard deviation, and categorical variables are presented as counts and percentages. We assessed the association of the continuous measure of air pollutant exposure with CWV, expressed as a 5 μg/m^3^ increment for PM_2.5_, a 5 ppb increment for NO_2_, and a 3 ppb increment for O_3_, as previously reported for relevant air pollutant concentrations in developed countries [8, 9]. The associations were explored using 5 linear mixed models, each with random intercepts representing the effect of recruitment centre. This random intercept was a proxy for spatial clustering of participants. We considered a parsimonious set of demographic, lifestyle, and environmental characteristics as potential covariates in the association between CWV and air pollution, considering collinearity. Our final variable selection was guided by published literature, previous knowledge, as well as variables that we observed to be effect modifiers of observed associations. Neighbourhood greenness did not meet the threshold for covariate selection and neighbourhood noise could not be tested; however, prior studies with noise adjustment showed stable if not larger effect estimates of association [9, 10]. The following fixed covariates were included in each model: 1) none (“unadjusted model”); 2) participant’s age, sex, and ethnicity (“basic model”); 3) further adjusted for contributing individual-level factors *i*.*e*., the INTERHEART risk score, education, and working outside of the lived-in community (“lifestyle model”); **4) further adjusted for community-level factors i.e. walkability, material factor score, and social factor score (our a priori “primary model”)** and 5) model further adjusted for co-pollutants within two-pollutant models at a time (“co-pollutant model”). A complete case analysis was employed because missing data on covariates was low (1.6% for education and 0.08% for Can-ALE index). In sensitivity analyses, models 1–5 were i. stratified by sex and ii. repeated after excluding participants based on immigration status for those who had been in Canada for less than ten years (n = 5885), and iii. stratified based on workplace location (residing at home/working in the lived-in community versus working outside the lived-in community) to address possible exposure misclassification due to major time away from residence. To investigate interactions between the pollutants, we modeled the effect of one pollutant on CWV within low, medium, and high levels of a second pollutant with the same fixed covariates of models 1–4 and tested the statistical significance of the interaction term of the two pollutants. A 2-sided p <0.05 was considered nominally significant with no adjustment for multiple testing. All analyses were completed using SAS software, version 9.4 (SAS Institute Inc).

## Results

### Participant characteristics

Of the 6645 participants enrolled in this study, 3253 (48.9%) were from Ontario, 1575 (23.7%) from Quebec, 744 (11.2%) from British Columbia, 671 (10.1%) from Nova Scotia, and 402 (6.0%) from Alberta. For all the regions, over 92% of the cohort’s postal codes were in urban areas. The mean age of participants at enrolment was 57.6 years (SD = 8.8; range = 32–81 years) and 56.0% of participants were women. The mean CWV of participants was 900.1 mm^3^ (165.1) at the time of the MRI scan. Demographic, anthropometric, and lifestyle characteristics of the study participants are found in Tables 1 and 2. The mean (SD) 5-year pollutant concentrations immediately preceding enrolment for PM_2.5_ was 6.9 μg/m^3^ (2.0), ranging from the lowest [3.2 μg/m^3^ (0.5)] in parts of the Calgary, Alberta region to the highest [8.6 μg/m^3^ (1.5)] in London, Ontario; for NO_2_ was 12.9 ppb (5.9), ranging from lowest [4.1 ppb (1.2)] in Halifax, Nova Scotia to highest [17.0 ppb (3.9)] in Toronto, Ontario; and for O_3_ was 24.6 ppb (4.0), ranging from lowest [16.9 ppb (3.0)] in Vancouver, British Columbia to highest [30.4 ppb (1.2)] in London, Ontario, as presented in Table 3. Participant characteristics stratified by sex are presented in S1–S3 Tables.

**Table 1:** Anthropometric characteristics of the study population by region of Canada.

			Region of Canada				
	N	Overall	BC	AB	ON	QC	NS
Number of participants	6645	6645	744	402	3253	1575	671
Women, n (%)	6645	3718 (56.0)	408 (54.8)	197 (49.0)	1948 (59.9)	811 (51.5)	354 (52.8)
Age, y	6645	57.6 (8.8)	56.8 (8.8)	53.3 (8.8)	57.5 (9.0)	58.6 (7.8)	59.0 (9.3)
Weight, kg	6645	76.3 (16.5)	72.5 (15.9)	80.7 (17.5)	75.7 (16.5)	77.4 (16.4)	78.1 (15.4)
Height, cm	6645	168.5 (9.4)	167.5 (9.6)	173.2 (10.1)	168.3 (9.1)	167.6 (9.3)	169.4 (9.2)
**Body Mass Index, mean (SD), kg/m** ^**2**^	6645	26.8 (4.9)	25.7 (4.4)	26.8 (4.9)	26.6 (4.9)	27.5 (5.0)	27.2 (4.8)
<25 (Normal), n (%)	6645	2657 (40.0)	352 (47.3)	163 (40.5)	1366 (42.0)	535 (34.0)	241 (35.9)
25–29 (Overweight), n (%)	6645	2536 (38.2)	285 (38.3)	150 (37.3)	1193 (36.7)	647 (41.1)	261 (38.9)
30+ (Obese), n (%)	6645	1452 (21.9)	107 (14.4)	89 (22.1)	694 (21.3)	393 (25.0)	169 (25.2)
Percent Body Fat, %	6624	30.6 (9.2)	28.9 (8.4)	29.9 (9.1)	30.9 (9.4)	31.3 (8.8)	30.0 (9.6)
Waist, cm	6645	88.6 (13.7)	84.3 (12.8)	91.0 (13.9)	87.5 (13.6)	90.3 (14.0)	93.0 (12.5)
Hip, cm	6645	101.3 (10.7)	99.2 (9.2)	103.6 (10.3)	100.4 (11.5)	102.3 (9.7)	104.7 (9.2)
Waist to hip ratio	6645	0.87 (0.08)	0.85 (0.09)	0.88 (0.08)	0.87 (0.08)	0.88 (0.09)	0.89 (0.08)
Waist circumference obese, n (%)	6645	1924 (29.0)	118 (15.9)	130 (32.3)	917 (28.2)	492 (31.2)	267 (39.8)
**Blood Pressure**							
Systolic, mmHg	6645	129 (17)	126 (17)	127 (14)	128 (17)	131 (16)	133 (16)
Diastolic, mmHg	6645	80 (10)	80 (10)	85 (9)	78 (10)	80 (9)	80 (10)
Heart rate, beats/minute	6644	70.4 (11.0)	69.4 (11.0)	70.5 (11.0)	71.1 (11.1)	69.7 (10.7)	69.6 (10.6)
**MRI-measured Outcomes**							
Carotid wall volume, mm^3^	6645	900.1 (165.1)	897.9 (170.5)	889.9 (170.5)	906.0 (163.2)	893.2 (155.8)	896.2 (184.3)

Presented data are means (SD) unless otherwise indicated.

**Table 2:** Demographics & lifestyle characteristics of the study population by region of Canada.

			Region of Canada				
	N	Overall	BC	AB	ON	QC	NS
Number of participants	6645	6645	744	402	3253	1575	671
Women	6645	3718 (56.0)	408 (54.8)	197 (49.0)	1948 (59.9)	811 (51.5)	354 (52.8)
Age, mean (SD), y	6645	57.6 (8.8)	56.8 (8.8)	53.3 (8.8)	57.5 (9.0)	58.6 (7.8)	59.0 (9.3)
**Self-reported ethnicity**							
East & South East Asian	6645	894 (13.5)	282 (37.9)	14 (3.5)	585 (18.0)	8 (0.5)	5 (0.7)
South Asian	6645	223 (3.4)	67 (9.0)	3 (0.7)	148 (4.5)	1 (0.1)	4 (0.6)
White	6645	5387 (81.1)	364 (48.9)	381 (94.8)	2449 (75.3)	1545 (98.1)	648 (96.6)
Other^a^	6645	141 (2.1)	31 (4.2)	4 (1.0)	71 (2.2)	21 (1.3)	14 (2.1)
**Highest Education Attained**							
High school or less	6541	842 (12.9)	100 (13.5)	44 (10.9)	344 (10.6)	290 (18.4)	64 (10.9)
College or Trade	6541	2107 (32.2)	255 (34.5)	117 (29.1)	891 (27.5)	671 (42.6)	173 (29.4)
University Degree	6541	3592 (54.9)	385 (52.0)	241 (60.0)	2001 (61.8)	613 (38.9)	352 (59.8)
**Smoke status**							
Current	6645	352 (5.3)	29 (3.9)	18 (4.5)	156 (4.8)	119 (7.6)	30 (4.5)
Former	6645	2241 (33.7)	178 (23.9)	132 (32.8)	971 (29.8)	732 (46.5)	228 (34.0)
Never	6645	4052 (61.0)	537 (72.2)	252 (62.7)	2126 (65.4)	724 (46.0)	413 (61.5)
**Living with partner/married**	6537	4938 (75.5)	567 (76.6)	314 (78.1)	2431 (75.2)	1141 (72.4)	485 (82.5)
**Employment**							
Full or part time	6534	4624 (70.8)	554 (74.7)	324 (80.6)	2208 (68.2)	1124 (71.5)	414 (71.4)
Retired	6534	1436 (22.0)	143 (19.3)	41 (10.2)	727 (22.5)	385 (24.5)	140 (24.1)
No paid work	6534	474 (7.3)	45 (6.1)	37 (9.2)	302 (9.3)	64 (4.1)	26 (4.5)
**Individual Social disadvantage score**							
Social disadvantage Index, mean (SD)	6121	1.2 (1.3)	1.2 (1.3)	0.8 (1.1)	1.2 (1.3)	1.4 (1.3)	1.1 (1.3)
Low disadvantage	6121	3677 (60.1)	435 (63.0)	283 (73.7)	1758 (59.1)	858 (56.9)	343 (61.0)
Moderate disadvantage	6121	2072 (33.9)	209 (30.3)	92 (24.0)	1031 (34.6)	542 (35.9)	198 (35.2)
High disadvantage	6121	372 (6.1)	46 (6.7)	9 (2.3)	187 (6.3)	109 (7.2)	21 (3.7)
**INTERHEART risk score** mean (SD)	6645	10.0 (5.7)	9.2 (5.5)	9.6 (5.7)	10.1 (5.7)	10.3 (5.8)	10.2 (5.6)
Low	6645	3392 (51.0)	418 (56.2)	217 (54.0)	1645 (50.6)	785 (49.8)	327 (48.7)
Moderate	6645	2123 (31.9)	223 (30.0)	121 (30.1)	1069 (32.9)	491 (31.2)	219 (32.6)
High	6645	1130 (17.0)	103 (13.8)	64 (15.9)	539 (16.6)	299 (19.0)	125 (18.6)
**Usual workplace location**							
Outside home community	6605	2301 (34.8)	269 (36.5)	197 (49.0)	1008 (31.1)	675 (43.3)	152 (22.8)

Presented data are n (%) unless otherwise specified.

^a^ Includes Blacks, Indigenous, Mixed and unknown ethnicity

**Table 3:** Environmental characteristics of the study population by region of Canada.

			Region of Canada				
	N	Overall	BC	AB	ON	QC	NS
Number of participants	6645	6645	744	402	3253	1575	671
Urban postal code	6645	6390 (96.2)	686 (92.2)	389 (96.8)	3179 (97.7)	1468 (93.2)	668 (99.6)
**Neighbourhood Deprivation**							
Material factor score	6447	-0.017 (0.041)	-0.013 (0.039)	-0.040 (0.035)	-0.016 (0.043)	-0.008 (0.039)	-0.027 (0.032)
Social factor score	6447	0.001 (0.041)	-0.006 (0.036)	-0.008 (0.044)	-0.005 (0.042)	0.017 (0.036)	0.008 (0.038)
**Walkability Measures**							
ALE Index	6640	1.806 (4.501)	1.535 (3.661)	0.810 (1.854)	2.618 (5.610)	1.172 (2.976)	0.255 (1.764)
ALE Index Class, n (%)							
Class 1: very low	6640	772 (11.6)	81 (10.9)	40 (10.0)	300 (9.2)	232 (14.7)	119 (17.7)
Class 2	6640	1917 (28.9)	176 (23.7)	193 (48.0)	820 (25.2)	472 (30.0)	256 (38.2)
Class 3	6640	2014 (30.3)	249 (33.6)	146 (36.3)	1079 (33.2)	326 (20.7)	214 (31.9)
Class 4	6640	1182 (17.8)	166 (22.4)	12 (3.0)	611 (18.8)	311 (19.8)	82 (12.2)
Class 5: very high	6640	755 (11.4)	70 (9.4)	11 (2.7)	442 (13.6)	232 (14.7)	0 (0.0)
**Linked Air Quality Measures**							
PM_2.5_, ug/m^3^, over 2008–2012	6645	6.9 (2.0)	6.7 (1.4)	3.2 (0.5)	8.2 (1.5)	6.0 (1.5)	5.2 (1.1)
NO_2_, ppb, over 2008–2012	6645	12.9 (5.9)	15.2 (4.8)	12.8 (3.5)	14.1 (5.3)	13.0 (5.8)	4.1 (1.2)
O_3_, ppb, over 2008–2012	6645	24.6 (4.0)	17.8 (4.3)	23.4 (2.1)	27.1 (2.6)	23.9 (2.4)	22.3 (1.3)

Presented data are means (SD) unless otherwise indicated. CMA: census metropolitan area; ALE: active living environment

### Long-term pollutant exposure and MRI-measured CWV–Primary analysis

**Fig 1:**
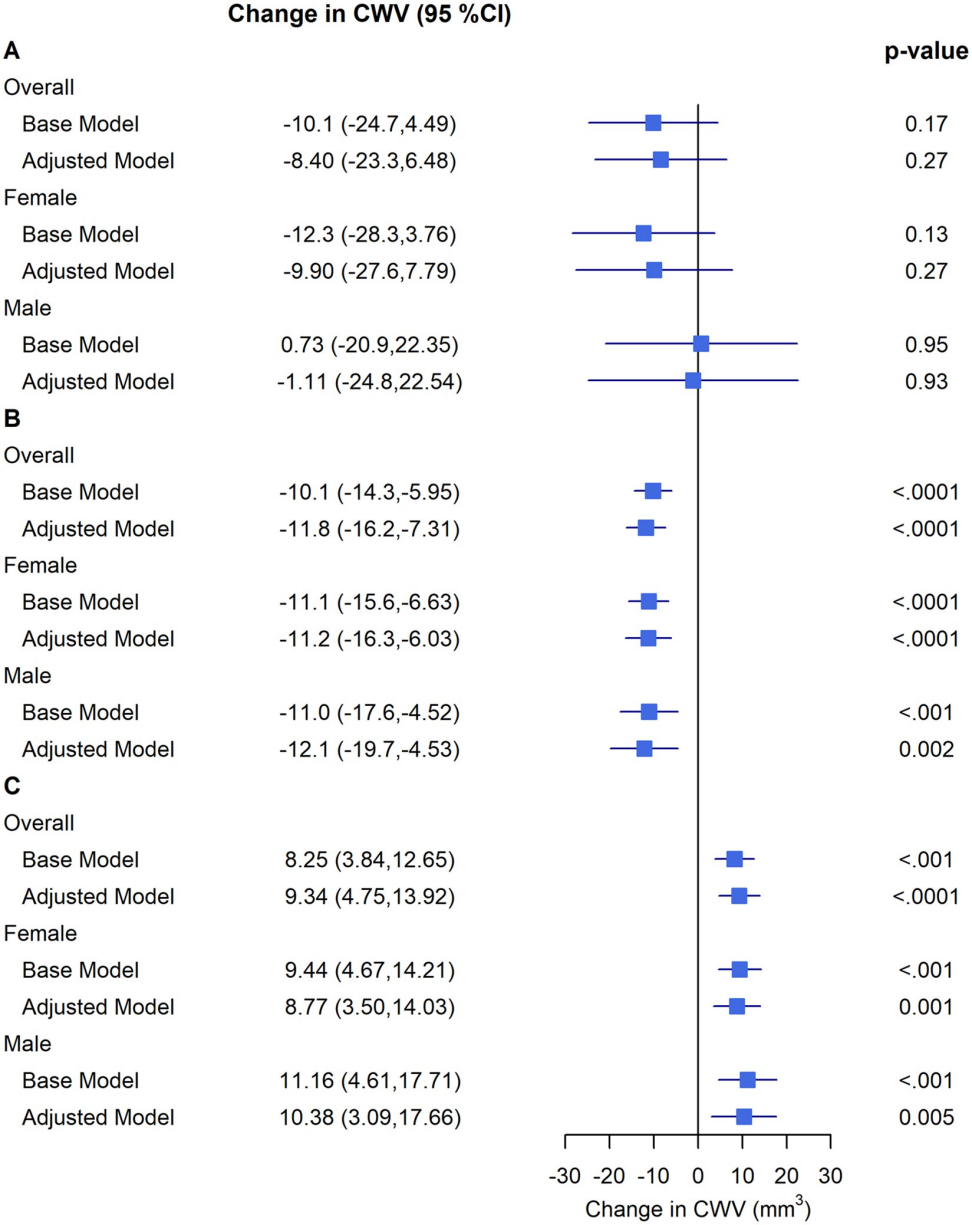
Associations of 5-year pollutant exposures with subclinical atherosclerosis as measured by carotid wall volume (CWV). Presented data are carotid wall volume adjusted estimates (95% CI) per (A) 5 μg/m^3^ increase for PM_2.5_, (B) 5 ppb increase for NO_2_, and (C) 3 ppb increase for O_3_, from a linear mixed model with centre modelled as a random intercept. The base model has no fixed effects; the adjusted model includes age, (sex), ethnicity, INTERHEART risk score, education, workplace location, walkability categories, material factor score, and social factor score.

Associations of 5-year pollutant exposures with subclinical atherosclerosis as measured by CWV are presented in Fig 1 and Table 4. The association between PM_2.5_ and CWV was inconsistent and not statistically significant in the primary model. A 5 μg/m^3^ higher PM_2.5_ concentration was not associated with CWV (mean = -8.4 mm^3^; 95% CI -23.2, 6.48; p = 0.27). A 5 ppb higher NO_2_ concentration was associated with 11.8 mm^3^ lower CWV (95% CI -16.2, -7.31; p<0.0001); contradictory to our hypothesis. A 3 ppb increase in O_3_ was associated with 9.34 mm^3^ higher CWV (95% CI 4.75, 13.92; p<0.0001). These results remained consistent in the sensitivity analysis in males and females, after excluding immigrants with less than 10 years of residence in Canada, and after excluding participants working away from their lived-in community (Fig 1, Tables 4 and 5). Of note, Pearson correlation between PM_2·5_ and NO_2_ was [r = +0·39; p<·0001], between PM_2·5_ and O_3_ was [r = +0·16; p<·0001], and between NO_2_ and O_3_ was [r = -0·23; p<·0001].

**Table 4:** Association of carotid wall volume (mm^3^) with pollutants exposure.

	Overall (n = 6223)		Females (n = 3472)		Males (n = 2751)		Remove recent immigrants (n = 5885)	
	Effect (95% CI)	*p-*	Effect (95% CI)	*p-*	Effect (95% CI)	*p-*	Effect (95% CI)	*p-*
**PM**_**2.5**_								
Model 1	-10.1 (-24.7,4.49)	0.1747	-12.3 (-28.3,3.76)	0.1333	0.73 (-20.9,22.35)	0.9469	-12.5 (-27.6,2.71)	0.1075
Model 2	-14.9 (-28.4,-1.42)	0.0303	-17.9 (-33.9,-1.87)	0.0286	-6.46 (-28.2,15.32)	0.5610	-18.2 (-32.1,-4.21)	0.0107
Model 3	-15.0 (-28.5,-1.55)	0.0288	-18.5 (-34.5,-2.46)	0.0238	-6.18 (-27.9,15.58)	0.5777	-18.4 (-32.3,-4.41)	0.0099
Model 4	-8.40 (-23.3,6.48)	0.2685	-9.90 (-27.6,7.79)	0.2728	-1.11 (-24.8,22.54)	0.9269	-12.5 (-27.9,2.96)	0.1130
Model 5:+NO_2_	2.26 (-13.2,17.70)	0.7747	0.94 (-17.5,19.37)	0.9207	8.84 (-15.6,33.23)	0.4775	-2.32 (-18.3,13.66)	0.7762
Model 5:+O_3_	0.60 (-14.7,15.91)	0.9391	-1.35 (-19.2,16.46)	0.8818	6.28 (-17.9,30.45)	0.6103	-2.49 (-18.3,13.31)	0.7577
**NO**_**2**_								
Model 1	-10.1 (-14.3,-5.95)	<.0001	-11.1 (-15.6,-6.63)	<.0001	-11.0 (-17.6,-4.52)	0.0009	-10.2 (-14.5,-5.89)	<.0001
Model 2	-11.2 (-15.0,-7.32)	<.0001	-11.1 (-15.6,-6.66)	<.0001	-10.7 (-17.2,-4.14)	0.0014	-11.2 (-15.2,-7.25)	<.0001
Model 3	-11.0 (-14.9,-7.10)	<.0001	-11.6 (-16.1,-7.08)	<.0001	-9.96 (-16.5,-3.38)	0.0030	-11.0 (-15.0,-7.02)	<.0001
Model 4	-11.8 (-16.2,-7.31)	<.0001	-11.2 (-16.3,-6.03)	<.0001	-12.1 (-19.7,-4.53)	0.0018	-11.9 (-16.5,-7.37)	<.0001
Model 5:+O_3_	-9.66 (-14.8,-4.55)	0.0002	-9.42 (-15.4,-3.47)	0.0019	-9.13 (-17.5,-0.76)	0.0326	-8.85 (-14.1,-3.60)	0.0010
Model 5:+PM_2.5_	-11.9 (-16.5,-7.32)	<.0001	-11.2 (-16.6,-5.88)	<.0001	-12.8 (-20.6,-4.96)	0.0014	-11.8 (-16.5,-7.04)	<.0001
**O**_**3**_								
Model 1	8.25 (3.84,12.65)	0.0002	9.44 (4.67,14.21)	0.0001	11.16 (4.61,17.71)	0.0008	9.26 (4.76,13.76)	<.0001
Model 2	10.45 (6.34,14.55)	<.0001	9.86 (5.13,14.59)	<.0001	11.01 (4.41,17.60)	0.0011	11.59 (7.41,15.77)	<.0001
Model 3	10.36 (6.23,14.48)	<.0001	10.27 (5.52,15.02)	<.0001	10.44 (3.82,17.05)	0.0020	11.49 (7.29,15.69)	<.0001
Model 4	9.34 (4.75,13.92)	<.0001	8.77 (3.50,14.03)	0.0011	10.38 (3.09,17.66)	0.0053	10.82 (6.14,15.50)	<.0001
Model 5:+NO_2_	4.27 (-1.06,9.60)	0.1167	3.47 (-2.76,9.69)	0.2746	6.66 (-1.39,14.70)	0.1047	6.25 (0.82,11.68)	0.0241
Model 5:+PM_2.5_	9.39 (4.62,14.15)	0.0001	8.64 (3.22,14.06)	0.0018	10.81 (3.34,18.28)	0.0046	10.62 (5.77,15.47)	<.0001

Presented data are adjusted estimates (95% CI) per 5 μg/m^3^ increase for PM_2.5_, 5 ppb increase for NO_2_, and 3 ppb increase for O_3_, from a linear mixed model with centre modelled as a random intercept. Model 1, unadjusted. Model 2, adjusted for age, (sex), and ethnicity. Model 3 further adjusted for the INTERHEART risk score and education and workplace location. Primary model 4 further adjusted for walkability categories, material factor score and social factor score. Model 5 further adjusted for co-pollutants.

**Table 5:** Association of carotid wall volume (mm^3^) with pollutants exposure stratified by workplace location.

	Overall (n = 6223)		Working away (n = 2188)		Work in community (n = 4035)	
	Effect (95% CI)	*p-*	Effect (95% CI)	*p-*	Effect (95% CI)	*p-*
**PM**_**2.5**_						
Model 1	-10.1 (-24.7,4.49)	0.1747	-1.27 (-24.7,22.21)	0.9157	-13.2 (-31.1,4.67)	0.1476
Model 2	-14.9 (-28.4,-1.42)	0.0303	-5.83 (-27.8,16.16)	0.6034	-16.3 (-32.9,0.25)	0.0536
Model 3	-15.0 (-28.5,-1.55)	0.0288	-5.50 (-27.5,16.44)	0.6229	-16.8 (-33.4,-0.20)	0.0473
Model 4	-8.40 (-23.3,6.48)	0.2685	0.87 (-22.8,24.55)	0.9429	-9.81 (-28.3,8.64)	0.2974
Model 5:+NO_2_	2.26 (-13.2,17.70)	0.7747	3.77 (-20.8,28.29)	0.7630	5.21 (-14.0,24.40)	0.5944
Model 5:+O_3_	0.60 (-14.7,15.91)	0.9391	5.53 (-18.2,29.26)	0.6477	0.67 (-18.1,19.40)	0.9441
**NO**_**2**_						
Model 1	-10.1 (-14.3,-5.95)	<.0001	-3.62 (-10.6,3.31)	0.3059	-13.1 (-18.2,-7.93)	<.0001
Model 2	-11.2 (-15.0,-7.32)	<.0001	-4.35 (-10.8,2.06)	0.1833	-14.4 (-19.1,-9.64)	<.0001
Model 3	-11.0 (-14.9,-7.10)	<.0001	-3.62 (-10.1,2.82)	0.2702	-14.5 (-19.3,-9.69)	<.0001
Model 4	-11.8 (-16.2,-7.31)	<.0001	-3.40 (-10.9,4.15)	0.3771	-15.5 (-21.0,-10.1)	<.0001
Model 5:+O_3_	-9.66 (-14.8,-4.55)	0.0002	-1.64 (-9.81,6.52)	0.6927	-12.9 (-19.2,-6.53)	<.0001
Model 5:+PM_2.5_	-11.9 (-16.5,-7.32)	<.0001	-3.70 (-11.5,4.10)	0.3524	-16.0 (-21.6,-10.3)	<.0001
**O**_**3**_						
Model 1	8.25 (3.84,12.65)	0.0002	5.98 (-1.20,13.17)	0.1023	9.52 (4.29,14.75)	0.0004
Model 2	10.45 (6.34,14.55)	<.0001	6.65 (-0.07,13.37)	0.0524	12.58 (7.69,17.46)	<.0001
Model 3	10.36 (6.23,14.48)	<.0001	6.27 (-0.45,12.98)	0.0675	12.64 (7.72,17.56)	<.0001
Model 4	9.34 (4.75,13.92)	<.0001	4.93 (-2.42,12.27)	0.1884	11.92 (6.42,17.43)	<.0001
Model 5:+NO_2_	4.27 (-1.06,9.60)	0.1167	4.14 (-3.93,12.20)	0.3146	5.16 (-1.33,11.65)	0.1191
Model 5:+PM_2.5_	9.39 (4.62,14.15)	0.0001	5.34 (-2.15,12.83)	0.1622	11.97 (6.31,17.63)	<.0001

Presented data are adjusted estimates (95% CI) per 5 μg/m^3^ increase for PM_2.5_, 5 ppb increase for NO_2_, and 3 ppb increase for O_3_, from a linear mixed model with centre modelled as a random intercept. Model 1, unadjusted. Model 2, adjusted for age, sex, and ethnicity. Model 3 further adjusted for the INTERHEART risk score and education. Primary model 4 further adjusted for walkability categories, material factor score and social factor score. Model 5 further adjusted for co-pollutants.

### Effect of co-pollutant interactions on CWV

**Fig 2:**
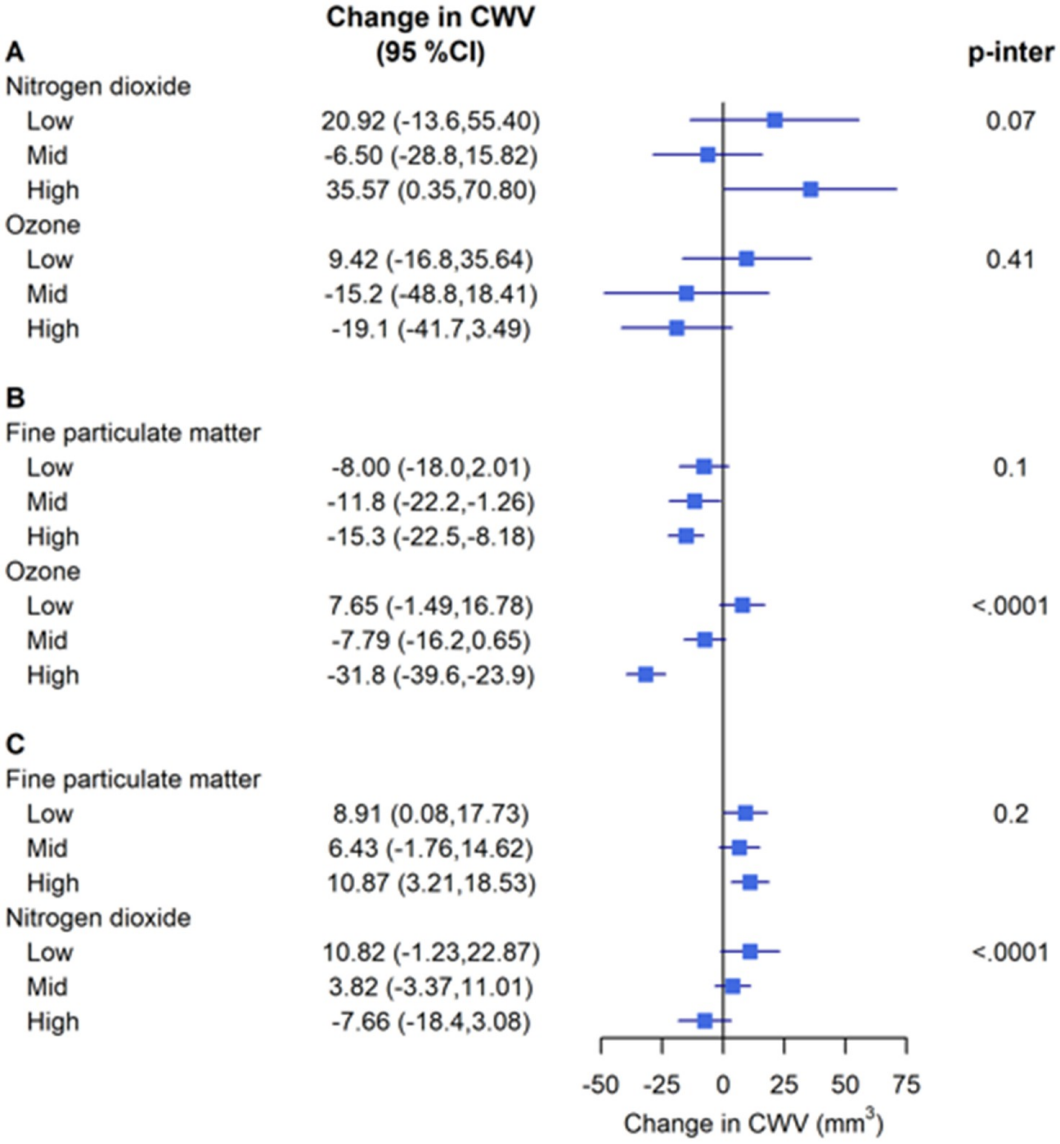
Effect of copollutant interactions on carotid wall volume (CWV). Presented data are carotid wall volume adjusted estimates (95% CI) per (A) 5 μg/m^3^ increase for PM_2.5_, (B) 5 ppb increase for NO_2_, and (C) 3 ppb increase for O_3_, from a linear mixed model with centre modelled as a random intercept and the following fixed effects: age, sex, ethnicity, INTERHEART risk score, education, workplace location, walkability categories, material factor score, and social factor score. Each pollutant is represented within co-pollutant tertiles and a p-value of the interaction term of the two pollutants (p-inter).

Across the primary models summarized in Fig 2 for testing the effect of one pollutant on CWV within low, medium, and high levels of a second pollutant, there was no evidence of interaction between PM_2.5_ and O_3_ or between PM_2.5_ and NO_2_. However, the association of NO_2_ with CWV differed according to the exposure levels of O_3_ (p<0.0001 for interaction) and the association of O_3_ with CWV differed according to the NO_2_ levels (p<0.0001 for interaction). The positive association of O_3_ with CWV within low and medium levels of NO_2_ was not observed within high levels of NO_2_ and the inverse association of NO_2_ with CWV within medium and high levels of O_3_ was not observed within low levels of O_3_. Thus, the gaseous pollutants emerged as mutual effect modifiers (Tables 6–8).

**Table 6:** Association of carotid wall volume (mm^3^) with O_3_ within co-pollutant exposure tertiles.

	**Overall** **N = 6223**		**Low PM**_**2.5**_ **(1.78–5.64 μg/m** ^**3**^ **) N = 1903**		**Mid PM**_**2.5**_ **5.65–8.18 μg/m** ^**3**^ **N = 1898**		**High PM**_**2.5**_ **8.18–11.2 μg/m** ^**3**^ **N = 2422**	
	**Effect** **(95% CI)**	***p-***	**Effect** **(95% CI)**	***p-***	**Effect** **(95% CI)**	***p-***	**Effect** **(95% CI)**	***p-***
Model 1	8.25 (3.84,12.65)	0.0002	7.16 (-1.00,15.32)	0.0855	9.56 (2.26,16.85)	0.0103	8.31 (0.24,16.39)	0.0436
Model 2	10.45 (6.34,14.55)	<.0001	9.73 (2.20,17.26)	0.0113	7.52 (-0.35,15.40)	0.0612	12.99 (5.55,20.44)	0.0006
Model 3	10.36 (6.23,14.48)	<.0001	9.96 (2.39,17.54)	0.0100	7.51 (-0.39,15.41)	0.0625	11.95 (4.49,19.42)	0.0017
Model 4	9.34 (4.75,13.92)	<.0001	8.91 (0.08,17.73)	0.0480	6.43 (-1.76,14.62)	0.1240	10.87 (3.21,18.53)	0.0055
			**Low NO**_**2**_ **(0.88–8.10 ppb)** **N = 1539**		**Mid NO**_**2**_ **(8.12–15.5 ppb)** **N = 2202**		**High NO**_**2**_ **(15.5–38.7 ppb)** **N = 2482**	
			**Effect** **(95% CI)**	***p-***	**Effect** **(95% CI)**	***p-***	**Effect** **(95% CI)**	***p-***
Model 1	8.25 (3.84,12.65)	0.0002	12.32 (0.91,23.74)	0.0343	0.56 (-7.10,8.21)	0.8869	-5.30 (-16.4,5.78)	0.3483
Model 2	10.45 (6.34,14.55)	<.0001	11.99 (0.90,23.08)	0.0341	4.21 (-3.01,11.42)	0.2533	-6.42 (-17.1,4.25)	0.2382
Model 3	10.36 (6.23,14.48)	<.0001	12.63 (1.55,23.70)	0.0255	4.28 (-2.93,11.48)	0.2443	-6.81 (-17.5,3.86)	0.2108
Model 4	9.34 (4.75,13.92)	<.0001	10.82 (-1.23,22.87)	0.0784	3.82 (-3.37,11.01)	0.2974	-7.66 (-18.4,3.08)	0.1622

Presented data are adjusted CWV estimates (95% CI) per, 3 ppb increase for O_3_, from a linear mixed model with centre modelled as a random intercept. Model 1, unadjusted. Model 2, adjusted for age, (sex), and ethnicity. Model 3 further adjusted for the INTERHEART risk score, education and workplace location. Primary model 4 further adjusted for walkability categories, material factor score and social factor score.

**Table 7:** Association of carotid wall volume (mm^3^) with PM_2.5_ within co-pollutant exposure tertiles.

	**Overall** **N = 6223**		**Low O**_**3**_ **14.2–22.9 ppb** **N = 1941**		**Mid O**_**3**_ **23.0–26.3 ppb** **N = 2296**		**High O**_**3**_ **26.3–39.1 ppb** **N = 1986**	
	**Effect** **(95% CI)**	***p-***	**Effect** **(95% CI)**	***p-***	**Effect** **(95% CI)**	***p-***	**Effect** **(95% CI)**	***p-***
Model 1	-10.1 (-24.7,4.49)	0.1747	5.51 (-14.6,25.63)	0.5911	-3.21 (-38.3,31.92)	0.8580	-20.5 (-40.9,-0.16)	0.0482
Model 2	-14.9 (-28.4,-1.42)	0.0303	5.89 (-19.8,31.54)	0.6527	-12.5 (-45.1,20.16)	0.4533	-21.7 (-40.5,-2.89)	0.0238
Model 3	-15.0 (-28.5,-1.55)	0.0288	6.90 (-18.8,32.64)	0.5990	-13.8 (-46.3,18.64)	0.4041	-21.9 (-40.8,-2.98)	0.0233
Model 4	-8.40 (-23.3,6.48)	0.2685	9.42 (-16.8,35.64)	0.4809	-15.2 (-48.8,18.41)	0.3756	-19.1 (-41.7,3.49)	0.0975
			**Low NO**_**2**_ **(0.88–8.10 ppb)** **N = 1539**		**Mid NO**_**2**_ **(8.12–15.5 ppb)** **N = 2202**		**High NO**_**2**_ **(15.5–38.7 ppb)** **N = 2482**	
			**Effect** **(95% CI)**	***p-***	**Effect** **(95% CI)**	***p-***	**Effect** **(95% CI)**	***p-***
Model 1	-10.1 (-24.7,4.49)	0.1747	24.21 (-10.3,58.67)	0.1684	1.51 (-20.3,23.36)	0.8922	31.73 (-4.58,68.04)	0.0867
Model 2	-14.9 (-28.4,-1.42)	0.0303	13.62 (-18.1,45.31)	0.3995	0.59 (-20.2,21.34)	0.9553	32.17 (-2.85,67.20)	0.0718
Model 3	-15.0 (-28.5,-1.55)	0.0288	12.68 (-19.2,44.60)	0.4360	0.18 (-20.6,20.96)	0.9862	33.37 (-1.54,68.27)	0.0610
Model 4	-8.40 (-23.3,6.48)	0.2685	20.92 (-13.6,55.40)	0.2342	-6.50 (-28.8,15.82)	0.5678	35.57 (0.35,70.80)	0.0478

Presented data are adjusted CWV estimates (95% CI) per 5 μg/m^3^ increase for PM_2.5_ from a linear mixed model with centre modelled as a random intercept. Model 1, unadjusted. Model 2, adjusted for age, (sex), and ethnicity. Model 3 further adjusted for the INTERHEART risk score, education and workplace location. Primary model 4 further adjusted for walkability categories, material factor score and social factor score.

**Table 8:** Association of carotid wall volume (mm^3^) with NO_2_ within co-pollutant exposure tertiles.

	**Overall** **N = 6223**		**Low O**_**3**_ **14.2–22.9 ppb** **N = 1941**		**Mid O**_**3**_ **23.0–26.3 ppb** **N = 2296**		**High O**_**3**_ **26.3–39.1 ppb** **N = 1986**	
	**Effect** **(95% CI)**	***p-***	**Effect** **(95% CI)**	***p-***	**Effect** **(95% CI)**	***p-***	**Effect** **(95% CI)**	***p-***
Model 1	-10.1 (-14.3,-5.95)	<.0001	0.44 (-5.33,6.21)	0.8821	-4.72 (-13.4,3.95)	0.2857	-27.5 (-35.1,-20.0)	<.0001
Model 2	-11.2 (-15.0,-7.32)	<.0001	6.95 (-1.29,15.18)	0.0981	-6.57 (-14.5,1.38)	0.1054	-27.7 (-34.6,-20.8)	<.0001
Model 3	-11.0 (-14.9,-7.10)	<.0001	7.06 (-1.19,15.31)	0.0935	-5.80 (-13.7,2.13)	0.1514	-28.0 (-34.9,-21.0)	<.0001
Model 4	-11.8 (-16.2,-7.31)	<.0001	7.65 (-1.49,16.78)	0.1008	-7.79 (-16.2,0.65)	0.0706	-31.8 (-39.6,-23.9)	<.0001
			**Low PM**_**2.5**_ **(1.78–5.64 μg/m** ^**3**^ **) N = 1903**		**Mid PM**_**2.5**_ **5.65–8.18 μg/m** ^**3**^ **N = 1898**		**High PM**_**2.5**_ **8.18–11.2 μg/m** ^**3**^ **N = 2422**	
			**Effect** **(95% CI)**	***p-***	**Effect** **(95% CI)**	***p-***	**Effect** **(95% CI)**	***p-***
Model 1	-10.1 (-14.3,-5.95)	<.0001	-5.36 (-14.4,3.68)	0.2450	-13.5 (-23.3,-3.61)	0.0074	-14.8 (-22.3,-7.29)	0.0001
Model 2	-11.2 (-15.0,-7.32)	<.0001	-7.41 (-15.8,0.96)	0.0828	-11.1 (-20.7,-1.61)	0.0219	-15.4 (-22.3,-8.59)	<.0001
Model 3	-11.0 (-14.9,-7.10)	<.0001	-7.68 (-16.1,0.77)	0.0749	-11.1 (-20.6,-1.46)	0.0239	-14.7 (-21.6,-7.73)	<.0001
Model 4	-11.8 (-16.2,-7.31)	<.0001	-8.00 (-18.0,2.01)	0.1173	-11.8 (-22.2,-1.26)	0.0281	-15.3 (-22.5,-8.18)	<.0001

Presented data are adjusted CWV estimates (95% CI) per 5 ppb increase for NO_2_ from a linear mixed model with centre modelled as a random intercept. Model 1, unadjusted. Model 2, adjusted for age, (sex), and ethnicity. Model 3 further adjusted for the INTERHEART risk score, education and workplace location. Primary model 4 further adjusted for walkability categories, material factor score and social factor score.

## Discussion

In this cohort study among 6645 Canadian healthy adults using spatially resolved pollutant concentrations and comprehensive covariate adjustments, long-term exposure to air pollution was inconsistently associated with CWV as measured by MRI. PM_2.5_ associations with CWV were inconsistent while NO_2_ was associated with decreased CWV; a finding that was contradictory to our expectation. Exposure to ground-level O_3_ was associated with increased CWV (noting the limitation that O_3_ resolution is 10 km). These findings were consistent between men and women and remained robust even after exclusion of recent immigrants. Lastly, NO_2_ and O_3_ mutually modified the associations of these pollutants with CWV.

There is limited evidence that direct biological measures of early CVD are influenced by exposure to air pollution at low levels. However, the MAPLE study and MAPLE phase 2 report associations between nonaccidental mortality and cardiovascular-related mortality and long-term exposure to ambient PM_2.5_ levels, including at concentrations below national air quality standards [19, 26]. The evidence is adequate for overall TRAP association with CVD mortality, but remains inconclusive for CVD morbidity *i*.*e*., the effect of air pollution on traditional cardiovascular risk factors [9, 27]. The 2022 HEI report concludes that additional studies are needed for cardiometabolic outcomes and subclinical biomarkers [10].

Ultrasound measurement of cIMT is widely used because it is inexpensive, non-invasive, and does not involve exposure to a magnetic field, but it has limited clinical usefulness beyond traditional CVD risk factors [20]. Carotid wall thickness, determined by MRI, generates more reproducible measurements than ultrasound and is a superior measure of early plaque development [12, 13]—ideal for the investigation of healthy middle-aged populations. It is also more consistently associated with incident CVD than ultrasound-measured cIMT [14]. Compared to cIMT, CWV includes the adventitia, the source of vasa vasorum [12]. In terms of sensitivity of MRI-measured CWV, studies have suggested adventitial thickening to be an early sign of atherosclerosis, whereas a dense network of adventitial vasa vasorum can signify progression of atherosclerosis to symptomatic disease [12]. We have previously shown in CAHHM that simple cardiovascular risk scores were significantly associated with CWV, where mean (SD) CWV for low, medium, and high INTERHEART risk score categories were 881.5 (163.1), 915.4 (166.6), and 940.9 (172.9) mm^3^, respectively [11]. Therefore, the overall mean in the current analysis set (900 mm^3^) corresponds to someone with a low-moderate INTERHEART risk score. The inconsistent association between PM_2.5_ and a state-of-the-art surrogate marker for subclinical atherosclerosis we observed across Canada parallels previous findings of the Multicultural Community Health Assessment Trial (M-CHAT) study between multiple particle and gaseous measures of TRAP (e.g., NO_2_, PM_2.5_, black carbon) and progression of carotid artery atherosclerosis in Vancouver, BC.^6^ This suggests that PM_2.5_ is not affecting CV risk through early atherosclerosis formation. Therefore, if there is an increased risk with exposure to PM_2.5_, it may operate through other intermediary pathways such as release of proinflammatory mediators, autonomic nervous system perturbations, and translocation of particle constituents into the blood, acting independently from the promotion of plaque build-up [28]. We therefore recommend employing CWV over cIMT, especially in relatively low exposure settings such as Canada where a more precise measurement of subclinical atherosclerosis may be of higher yield. Of note, walkability was shown to modulate the effects of PM_2.5_ in the main and secondary analyses; a neighbourhood characteristic that has been scarcely captured in previous literature.

The weak inverse association of NO_2_ (an indicator of TRAP and/or combustion pollution in general) with CWV in our study is unexpected and needs careful evaluation. There is insufficient collective evidence in the literature linking NO_2_ or TRAP with subclinical atherosclerosis in healthy adults specifically. The evidence of an association between TRAP and cardiovascular morbidity is low [9]. Three of 5 studies (n = 144,787) included in a meta-analysis of NO_2_ and ischemic heart disease (IHD) [10] incidence showed a positive association (a prospective cohort conducted across 11 European cohorts [the European Study of Cohorts for Air Pollution Effects: ESCAPE] [29]; Athens, Greece [30]; and Oakland, California [31]), while two studies (n = 663,751) reported a negative association (London, UK [32]; Vancouver, BC [6]). In fact, the meta-analytical summary estimate, relative risk of IHD events per 10 μg/m^3^ NO_2_, was 0.99 (0.94;1.05). MESA-Air did not find a relationship between NO_2_ or other pollutant exposures and cIMT change, instead exposure was positively associated with coronary artery calcification progression [2]. In four European cohort, ESCAPE findings were inconsistent for an association between NO_2_ and cIMT, in fact, all four cohorts and their meta-analytical estimate, showed an inverse association, similar to our observation in CAHHM [33]. However, in a study of a *high cardiovascular risk* population (2227 patients mean age of 62.9 years) in London, Ontario, NO_2_ exposure was associated with cumulative plaque burden as captured by carotid total plaque area (TPA) using two-dimensional ultrasound [34]. Collectively within the existing body of literature, it is plausible that NO_2_ is probably not involved in early carotid thickening but perhaps in more advanced morbid stages.

Our finding on the effect of chronic ambient O_3_ exposure on subclinical atherosclerosis is congruent with what the MESA study had previously reported on progression of IMT of the common carotid artery and new carotid plaque formation with outdoor O_3_ exposure in six U.S. city regions [8]. One underlying biochemical pathway might be through the formation of reactive oxygen species that further give rise to increased oxidative stress and persistent chronic systemic inflammation [8]. Yet, given the inverse association between O_3_ and NO_2_ generally in urban settings, this “effect” of O_3_ may simply reflect the inverse of the NO_2_ effect, and because the O_3_ spatial resolution is 10 km, more caution is needed. The Multicenter Ozone Study in oldEr Subjects (MOSES) found that controlled exposure to low-levels of O_3_ did not affect selected blood biomarkers of systemic inflammation and prothrombotic state (C-reactive protein, monocyte-platelet conjugates, and microparticle-associated tissue factor activity) [35].

Next, given the observed interaction between O_3_ and NO_2_, our study emphasizes the need for further investigation of different exposures in combination. It also emphasizes the value of adjusting for novel neighbourhood characteristics such as active living environment, to examine effect modification and help further investigate regional variability. Neighbourhood factors that might modify the association between air pollution and CWV include poverty/affluence, overcrowding, living in apartment buildings, commuting, and proximity to roads.

### Strengths and limitations

Our study has obtained unique health measurements of subclinical cardiovascular markers using MRI on nearly 6600 Canadians along with individual-level information on environmental factors and lifestyle known to influence cardiometabolic outcomes. While IMT has been criticized as an accurate marker of atherosclerosis with sensitivity limitations of the ultrasound methodology [34], our study uses MRI-characterized CWV to assess atherosclerosis. Moreover, compared to MESA which reported on cIMT progression in 3392 participants with low-exposure levels, our study is well-powered. Next, the cohort’s diverse geographic coverage across Canada offers an exposure gradient in ambient PM_2.5_, NO_2_, and O_3_ that parallels what has previously been explored by the national Canadian Census Health and Environment Cohort (CanCHEC) [19]. Finally, exposure to air pollution values represented a time frame prior to knowledge of the outcome for each participant, i.e., the air pollution data collected for the 5-year period prior to the MRI. Several limitations are important to mention. First, individual-level exposures were estimated based on residence address. While this is common in epidemiological air pollution studies, exposure misclassification is inevitable because in this study participants’ time away from residence and residential history were not taken into account in estimating long-term exposure to air pollutants. Second, the 5-year pollutant exposure period was fixed for all participants (2008–2012) regardless of when an individual’s enrolment occurred in the 2014–2018 window. Thus, the exposure window was not consistently 5-years prior to enrolment for all study participants (i.e., depending on the date of participant MRI scan, the 5-year window may lag behind the MRI by ~2 years if it was done in 2014, but by up to ~6 years if it was done in 2018), which further increases risk for exposure misclassification. However, studies have demonstrated temporal stability in the spatial patterns of air pollutants over 10 years, thus temporal variability in the exposure window relative to the enrollment date is not a significant source of uncertainty [36]. Because the causally relevant window for air pollution exposures remains unknown [37], future investigations are needed to examine varying exposure time-windows and lag periods. Moreover, the spatial resolution of O_3_ is 10 km, which may not be fine enough to capture exposure variability, may have led to a spurious association between O_3_ and CWV, or may have been confounded by suburban living. Third, because CAHHM is a prospective pan-Canadian cohort of cohorts across five provinces and participants were selected from existing cohorts, the sample is not a random sample of the Canadian population distribution, thereby limiting the generalizability of these findings. When compared to a cohort of adults who responded to the 2015 Canadian Community Health Survey, CAHHM participants were older, of higher socioeconomic status, but had a similar mean INTERHEART risk score [38]. This does not affect the exposure-to-outcome reliability of our results within CAHHM, but generalizability to younger populations and Canadians living outside major Canadian cities should be done with caution. Fourth, because of the small number of events in our cohort (n = 156 events (2.35%)), we were not powered to look at intraplaque hemorrhage. Lastly, as with any observational study, the risk of residual confounding (from factors such as diet, lifestyle factors, or pre-existing health conditions) cannot be excluded.

## Conclusion

In healthy adults living in clean or only mildly polluted environments, we found no consistent association between air pollution and atherosclerosis. Exposure to NO_2_ was negatively associated, and O_3_ was positively associated with CWV, a sensitive measure of subclinical atherosclerosis, while PM_2.5_ was not associated with CWV. These inconsistent results raise questions as to whether previous reports linking low-level exposure to air pollution and CVD morbidity may have suffered from uncontrolled confounding. The role of NO_2_ in atherosclerosis is complex and requires further investigation, as do combinations of exposure to air pollutants.

## Supporting information

S1 Fig Flow chart for Air pollution and MRI markers in CAHHM. (PDF)

S2 Fig Scatterplot of air pollutants by carotid wall volume measurements with regression lines stratified by sex. (PDF)

S1 Table Anthropometric characteristics of the study population by sex. (PDF)

S2 Table Demographics & lifestyle characteristics of the study population by sex. (PDF)

S3 Table Environmental characteristics of the study population by sex. (PDF)
