# Supplementary figures and images for: Exposure to air pollutants and subclinical carotid atherosclerosis measured by magnetic resonance imaging: A cross-sectional analysis
Source: PLoS One. 2024 Oct 31;19(10):e0309912. doi: 10.1371/journal.pone.0309912 (PMC11527219; doi:10.1371/journal.pone.0309912)

**Figure S1: Flow chart for Air pollution and MRI markers in CAHHM**

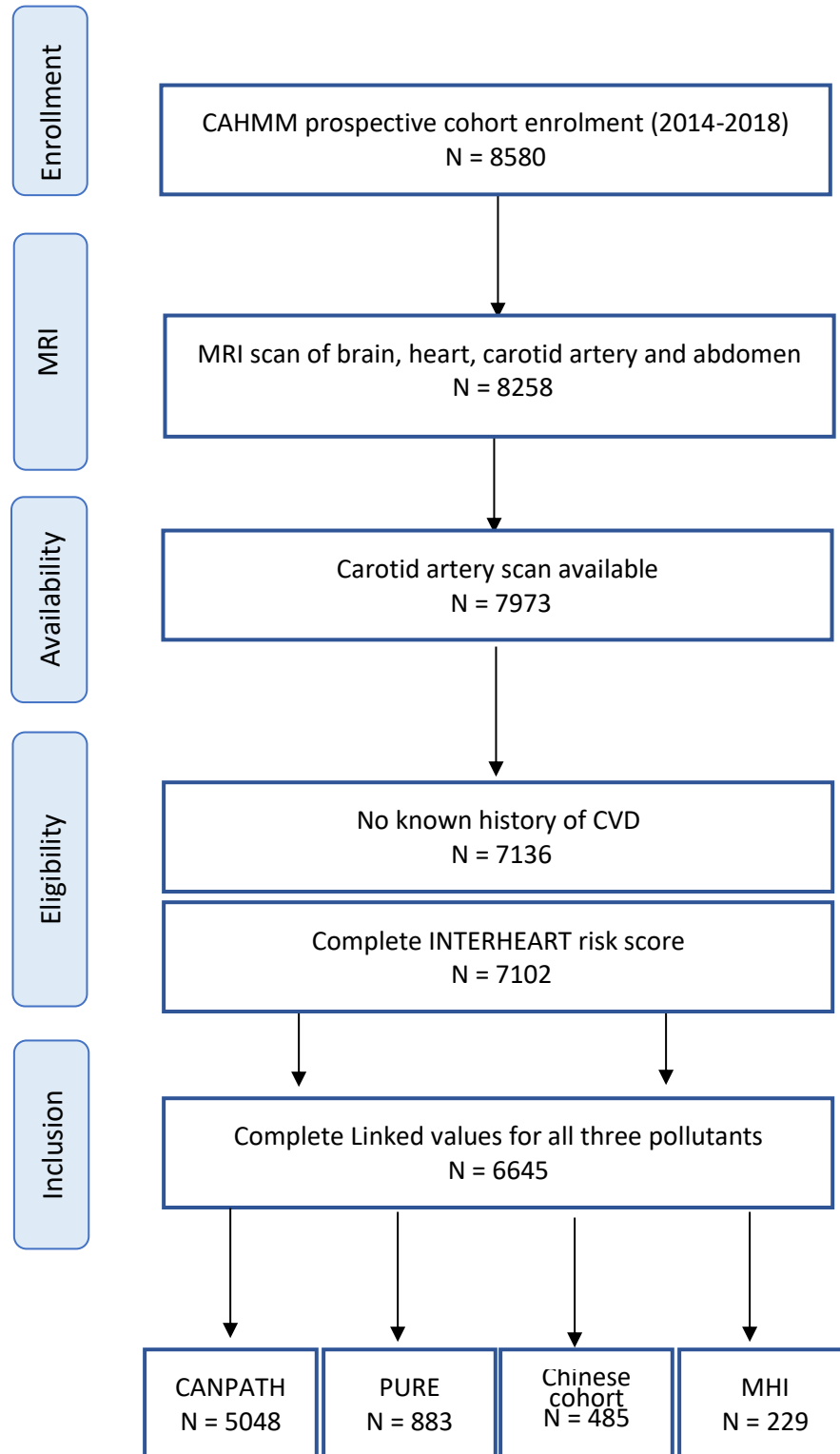

Supplement: S1 Fig — (PDF) [file pone.0309912.s001.pdf]
