# Supplementary material for: Exposure to air pollutants and subclinical carotid atherosclerosis measured by magnetic resonance imaging: A cross-sectional analysis
Source: PLoS One. 2024 Oct 31;19(10):e0309912. doi: 10.1371/journal.pone.0309912 (PMC11527219; doi:10.1371/journal.pone.0309912)
Supplement: S1 Table — (PDF) [file pone.0309912.s003.pdf]

Table S1. Anthropometric characteristics of the study population by sex

	N	Overall	Women	Men
Number of participants	6645	6645	3718	2927
Age, y	6645	57.6 (8.8)	57.1 (8.6)	58.1 (9.0)
Weight, kg	6645	76.3 (16.5)	70.0 (15.2)	84.3 (14.5)
Height, cm	6645	168.5 (9.4)	162.7 (6.6)	175.7 (7.1)
**Body Mass Index, mean (SD), kg/m^2**	6645	26.8 (4.9)	26.4 (5.4)	27.2 (4.2)
<25 (Normal), n (%)	6645	2657 (40.0)	1725 (46.4)	932 (31.8)
25-29 (Overweight), n (%)	6645	2536 (38.2)	1173 (31.5)	1363 (46.6)
30+ (Obese), n (%)	6645	1452 (21.9)	820 (22.1)	632 (21.6)
Percent Body Fat, %	6624	30.6 (9.2)	35.2 (8.1)	24.7 (6.9)
Waist, cm	6645	88.6 (13.7)	84.1 (13.5)	94.2 (11.9)
Hip, cm	6645	101.3 (10.7)	101.3 (12.2)	101.4 (8.4)
Waist to hip ratio	6645	0.87 (0.08)	0.83 (0.07)	0.93 (0.07)
Waist to hip ratio obese, n (%)	6645	3302 (49.7)	1388 (37.3)	1914 (65.4)
**Blood Pressure**				
Systolic, mmHg	6645	129 (17)	125 (17)	134 (15)
Diastolic, mmHg	6645	80 (10)	78 (10)	82 (10)
Heart rate, beats/minute	6644	70.4 (11.0)	71.3 (10.5)	69.3 (11.5)

Presented data are means (SD) unless otherwise indicated.
