# Supplementary material for: Exposure to air pollutants and subclinical carotid atherosclerosis measured by magnetic resonance imaging: A cross-sectional analysis
Source: PLoS One. 2024 Oct 31;19(10):e0309912. doi: 10.1371/journal.pone.0309912 (PMC11527219; doi:10.1371/journal.pone.0309912)
Supplement: S2 Table — (PDF) [file pone.0309912.s004.pdf]

Table S2. Demographics & Lifestyle characteristics of the study population by sex

	N	Overall	Women	Men
Number of participants	6645	6645	3718	2927
Age, mean (SD), y	6645	57.6 (8.8)	57.1 (8.6)	58.1 (9.0)
**Self-reported ethnicity**				
East & South East Asian	6645	894 (13.5)	517 (13.9)	377 (12.9)
South Asian	6645	223 (3.4)	95 (2.6)	128 (4.4)
White	6645	5387 (81.1)	3017 (81.1)	2370 (81.0)
Other[a]	6645	141 (2.1)	89 (2.4)	52 (1.8)
**Highest Education Attained**				
High school or less	6541	842 (12.9)	513 (14.0)	329 (11.4)
College or Trade	6541	2107 (32.2)	1225 (33.5)	882 (30.5)
University Degree	6541	3592 (54.9)	1915 (52.4)	1677 (58.1)
**Smoke status**				
Current	6645	352 (5.3)	189 (5.1)	163 (5.6)
Former	6645	2241 (33.7)	1225 (32.9)	1016 (34.7)
Never	6645	4052 (61.0)	2304 (62.0)	1748 (59.7)
Living with partner/married	6537	4938 (75.5)	2554 (69.9)	2384 (82.7)
**Employment**				
Full or part time	6534	4624 (70.8)	2540 (69.6)	2084 (72.3)
Retired	6534	1436 (22.0)	744 (20.4)	692 (24.0)
No paid work	6534	474 (7.3)	366 (10.0)	108 (3.7)
**Individual Social disadvantage score**				
Low disadvantage	6121	3677 (60.1)	1897 (56.1)	1780 (65.0)
Moderate disadvantage	6121	2072 (33.9)	1214 (35.9)	858 (31.3)
High disadvantage	6121	372 (6.1)	273 (8.1)	99 (3.6)
**INTERHEART risk score, mean (SD)**	6645	10.0 (5.7)	8.8 (5.4)	11.6 (5.8)
Low	6645	3392 (51.0)	2221 (59.7)	1171 (40.0)
Moderate	6645	2123 (31.9)	1063 (28.6)	1060 (36.2)
High	6645	1130 (17.0)	434 (11.7)	696 (23.8)
**Usual workplace location**				
Outside home community	6605	2301 (34.8)	1196 (32.3)	1105 (38.0)

Presented data are n (%) unless otherwise specified. [a] Includes Blacks, Indigenous, Mixed and unknown ethnicity.
