# Supplementary material for: Exposure to air pollutants and subclinical carotid atherosclerosis measured by magnetic resonance imaging: A cross-sectional analysis
Source: PLoS One. 2024 Oct 31;19(10):e0309912. doi: 10.1371/journal.pone.0309912 (PMC11527219; doi:10.1371/journal.pone.0309912)
Supplement: S3 Table — (PDF) [file pone.0309912.s005.pdf]

Table S3. Environmental characteristics of the study population by sex

	N	Overall	Women	Men
Number of participants	6645	6645	3718	2927
**Neighbourhood Deprivation**				
Material factor score	6447	-0.017 (0.041)	-0.017 (0.041)	-0.017 (0.040)
Social factor score	6447	0.001 (0.041)	0.002 (0.041)	-0.000 (0.041)
**Walkability Measures**				
ALE Index	6640	1.806 (4.501)	1.769 (4.320)	1.853 (4.721)
ALE Index Class, n (%)				
Class 1: very low	6640	772 (11.6)	436 (11.7)	336 (11.5)
Class 2	6640	1917 (28.9)	1037 (27.9)	880 (30.1)
Class 3	6640	2014 (30.3)	1156 (31.1)	858 (29.3)
Class 4	6640	1182 (17.8)	671 (18.1)	511 (17.5)
Class 5: very high	6640	755 (11.4)	414 (11.1)	341 (11.7)
**Number of participants in CMAs, n(%)**	6645	6134 (92.3)	3406 (91.6)	2728 (93.2)
**Linked Air Quality Measures**				
$PM_{2.5}$, ug/m^3, over 2008-2012	6645	6.9 (2.0)	7.0 (2.0)	6.7 (2.0)
NO_2, ppb, over 2008-2012	6645	12.9 (5.9)	12.9 (5.9)	12.9 (5.8)
Ozone, ppb, over 2008-2012	6645	24.6 (4.0)	24.8 (4.1)	24.3 (3.9)
Ozone warm season rolling 8hr average, ppb, over 2008-2012	1597	37.5 (7.7)	37.8 (7.7)	37.0 (7.6)

Presented data are means (SD) unless otherwise indicated.
