# Supplementary material for: Exposure to air pollutants and subclinical carotid atherosclerosis measured by magnetic resonance imaging: A cross-sectional analysis
Source: PLoS One. 2024 Oct 31;19(10):e0309912. doi: 10.1371/journal.pone.0309912 (PMC11527219; doi:10.1371/journal.pone.0309912)
Supplement: Attachment — Submitted filename: Response to Reviewers.docx [file pone.0309912.s006.docx]

28 September, 2023

Sabeena Jalal, MBBS, MSc, MSc, SM
Academic Editor
*PLOS ONE*

Response to initial response: [PONE-D-23-23654] ‘Ambient Air Pollution and Subclinical Carotid Atherosclerosis Measured by Magnetic Resonance Imaging: A Prospective Cohort Study’

Dear Dr. Jalal,

We are pleased to resubmit our manuscript to *PLOS ONE* following the 2^nd^ review. We have edited the manuscript and provide a point-by-point response to your comments. We feel based on your comments, and those from reviewers, that the manuscript quality has been strengthened and we look forward to your second review and response.

Please note that the editorial and reviewers’ comments are in *blue italics*, while our responses follow in normal font. We have tracked all changes made to the revised manuscript based on the reviewers’ comments in one copy of the manuscript.

Sincerely,

Russell de Souza, Sc.D., R.D.

Associate Professor, Population Genomics Program

Department of Health Research Methods, Evidence, and Impact

1200 Main St. West

MDCL, Room 3210

Hamilton, Ontario, Canada

L8N 3Z5

Phone (905) 525-9140 x. 22109

[desouzrj@mcmaster.ca](mailto:desouzrj@mcmaster.ca)

**Editor:**

*Dear Authors,*

*I have reviewed your manuscript detailing the association between air pollution and subclinical atherosclerosis in a Canadian cohort. Below, I outline both the strengths of your work and areas that require attention and revision.*

***Strengths:***

***Subject Matter:*** *The topic of your study is highly relevant and timely, addressing an important public health concern.*

***Methodology:*** *The use of MRI-characterized CWV to assess atherosclerosis is innovative and provides a more precise measurement.*

*Please address reviewer comments made by the two reviewers.*

***Furthermore, Areas for Revision:***

***Exposure Misclassification:*** *Please provide a more detailed explanation of how individual-level exposures were estimated based on residence address. Consideration of participants' time away from residence and residential history could enhance the accuracy of the exposure assessment.*

***Inconsistent Exposure Window:*** *The fixed 5-year pollutant exposure period for all participants may introduce bias. Please discuss this limitation and consider conducting sensitivity analyses to assess its impact.*

***Unexpected Findings:*** *The negative association of NO2 with CWV and the lack of association with PM2.5 were unexpected. A more comprehensive discussion of these findings and potential underlying factors is needed.*

***Resolution of O3 Data:*** *The spatial resolution of O3 data (10 km) may not be fine enough to capture local variations. Please discuss this limitation and its potential impact on the findings related to O3.*

***Lack of Consideration for Intraplaque Hemorrhage:*** *Please discuss the limitation of not being able to look at intraplaque hemorrhage and its potential significance in understanding the relationship between air pollution and atherosclerosis.*

***Additional Limitations:*** *Consider addressing other potential limitations such as selection bias, lack of longitudinal data, potential confounding variables, measurement error in pollution data, limited geographic scope, and potential interaction effects.*

*Your study has the potential to make a significant contribution to the field. However, the above concerns need to be addressed to enhance the robustness and credibility of the findings. I recommend a minor revision that includes a more detailed analysis of the exposure assessment, consideration of additional confounding variables, and a more comprehensive discussion of the unexpected findings and other limitations.*

We thank the **Editor** for recognizing the value of our work in cardiovascular disease and for sharing the reviewers’ viewpoints on our manuscript. We are confident that the raised points are adequately addressed in the revisions we have made as well as our detailed point-by-point responses below.

**Reviewer #1:**

*Hello,
The study presents valuable insights into the relationship between air pollution and subclinical atherosclerosis, utilizing a large sample size and advanced MRI techniques. The geographical diversity and comprehensive analysis add to the study's strengths. However, there are areas that require clarification and improvement:*

We thank **Reviewer 1** for taking the time to evaluate our manuscript, for the favourable response, and for the constructive feedback.

*1. Exposure Assessment: The authors should provide a more detailed explanation of the exposure misclassification risk and how the fixed 5-year pollutant exposure period might have affected the results. Clarifying these aspects would enhance the paper's transparency and allow readers to better assess the findings' validity.*

We thank **Reviewer 1** for pointing out the need for further clarity, a point also raised by **Reviewer 2**, that we shall address collectively here. We have now further elaborated on this limitation (Discussion, page 19) adding that “the exposure window was not consistently 5-years prior to enrolment for all study participants (i.e., depending on the date of participant MRI scan, the 5 year window may lag behind the MRI by ~2 years if it was done in 2014, but by up to ~6 years if it was done in 2018), which further increases risk for exposure misclassification” and that “future investigations are needed to examine varying exposure time-windows and lag periods.”

*2. Unexpected Findings: The unexpected negative association with NO2 and the lack of association with PM2.5 should be discussed more thoroughly. The authors should explore potential reasons for these findings and compare them more extensively with existing literature.*

We agree with both **Reviewer 1 and 2** that the negative association was surprising and unexpected. It is quite plausible that air pollution would not adversely affect all biological systems. Other MRI-measured outcome, including ectopic adipose tissue, silent vascular brain injury and brain volume, carotid atherosclerosis, left ventricular volumes, function and mass and silent myocardial infarction (MI) may not be associated with air pollution. We are currently examining some of these additional outcomes in work in progress. We have further discussed this point: “Collectively within the existing body of literature, it is plausible that NO_2_ is probably not involved in early carotid thickening but perhaps in more advanced morbid stages.”

We have also compared our NO_2_ results more extensively with existing literature (Discussion, page 18): “MESA-Air did not find a relationship between NO_2_ or other pollutant exposures and IMT change, instead exposure was positively associated with coronary artery calcification progression .^2^ In four European cohort, ESCAPE findings were inconsistent for an association between NO_2_ and cIMT, in fact, all four cohorts and their meta-analytical estimate, showed an inverse association, similar to our observation in CAHHM.” Please find below ESCAPE results summarized in Figure 1 of the paper.

Figure 1. Forest plot of the percent difference in CIMT (geometric mean with 95% CIs) for model M3 for (A) ESCAPE air pollutants per standard contrast of exposure as indicated in the figure. Source: Environmental Health Perspectives • volume 123 | number 6 | June 2015

*3. Resolution of O3 Data: The authors should discuss the limitations of the O3 spatial resolution and how it might have influenced the results.*

We agree with **Reviewer 1** that the resolution of O_3_ data is a limitation of our study. We have stated this limitation under methods and twice in the discussion. However, the same O_3_ data has been used in the MAPLE study (the major, recent air pollution and mortality work in Canada), where O_3_ was seen to be impacting mortality. Nevertheless, we have now added this limitation in the abstract and again in the last paragraph of the discussion (page 20), acknowledging the potential for a spurious association due to the difference in spatial resolution of this marker. More-resolved exposure estimates of O_3_ are very much needed for future work to better understand this, but this is complex because of the strong inverse correlation between O_3_ and NO_2_ as you go to finer and finer resolutions.

*4. Additional Analyses (Optional): If possible, further analyses could be conducted to address some of the limitations, such as considering participants' time away from residence or exploring the relationship with intraplaque hemorrhage.*

We thank **Reviewer 1** for this suggestion. We would like to point out that we have adjusted in our models for residing at home/working in the lived-in community versus working outside the lived-in community. We have now added to our sensitivity analyses, the stratified analysis based on this variable as a proxy for time away from residential address. These sensitivity analyses showed results consistent with our main analysis for all three pollutants for those working in the community (supplemental Table S5). As for intraplaque hemorrhage, we acknowledge that it is an important clinical/atherosclerosis outcome to investigate, however, in this cohort, prevalence of IPH (n=156 IPH events (2.35%)) is too low to be able to estimate this association reliably.

*5. The paper's contributions are significant, and the limitations do not undermine the overall value of the research. However, addressing these minor revisions would enhance the paper's clarity, coherence, and impact. Therefore, acceptance with minor revisions seems the most appropriate recommendation. Thank you.*

We would like to thank **Reviewer 1** for recognising the value of our work within the field. We feel based on your comments that the manuscript quality has been strengthened and we look forward to your second review and response.

*Reviewer #2:*

*Mention the limitations.*

*1. Selection Bias: If the study's participants were not randomly selected or if there was a lack of diversity in the sample (e.g., age, gender, geographic location), this could introduce selection bias, limiting the generalizability of the findings.*

We thank **Reviewer 2** for this comment. We have now added this limitation to the final paragraph of the discussion: “Third, because CAHHM is a prospective pan-Canadian cohort of cohorts across five provinces and participants were selected from existing cohorts, the sample is not a random sample of the Canadian population distribution, thereby limiting the generalizability of these findings. When compared to a cohort of adults who responded to the 2015 Canadian Community Health Survey, CAHHM participants were older, of higher socioeconomic status, but had a similar mean INTERHEART risk score.^38^ This does not affect the exposure-to-outcome reliability of our results within CAHHM, but generalizability to younger populations and Canadians living outside major Canadian cities should be done with caution.”

*2. Lack of Longitudinal Data: If the study was cross-sectional in nature, the lack of longitudinal data might hinder the ability to establish causal relationships between air pollution and atherosclerosis.*

We have elaborated on this limitation based on the Bradford Hill criteria for causality. “Exposure to air pollution values represented a time frame prior to knowledge of the outcome for each participant, i.e., the air pollution data collected for the 5-year period prior to the MRI. We are therefore comfortable describing this as a prospective association, despite the lack of longitudinal follow-up.”

*3. Potential Confounding Variables: If not all relevant confounding variables were controlled for, such as diet, lifestyle factors, or pre-existing health conditions, this could affect the validity of the associations found.*

We thank **Reviewer 2** for this point of consideration. We argue that a major strength of our study is the detailed and well-characterized phenotyping of our cohort. We have considered a parsimonious yet comprehensive set of confounders based on subject matter expertise and prior literature. These include the INTERHEART risk score that captures some dietary components, physical exercise, lifestyle and comorbidities summarizing individual cardiovascular risk. Nevertheless, we have now added to the limitations (Discussion, page 20) that “as with any observational study, the risk of residual confounding (from factors such as diet, lifestyle factors, or pre-existing health conditions) cannot be excluded.”

*4. Measurement Error in Pollution Data: If there were inaccuracies in the measurement of pollution levels, such as reliance on satellite data or modeling without sufficient ground-truthing, this could lead to misestimation of exposure levels.*

We thank **Reviewer 2** for this comment, however, as we mention under (Methods, page 7), “To adjust for any residual bias in the satellite-derived PM2.5 estimates, a geographically weighted regression (GWR) incorporating ground-based observations was then applied. Good agreement was found with cross-validated surface observations across North-America (R^2^ = 0.70).” This geographically-weighted regression uses the surface (ground-truth) PM2.5 to correct for bias in the satellite estimates resulting in an improved R^2^ so that the exposures (at least at the scale of North America as a whole) are not biased and the magnitude of the gradient (from lowest to highest exposure) is representative of what ground measurements show. Inevitably, there are limited numbers of ground stations, thus, there is still room for errors but we are using the best available estimates at this time.

*5. Lack of Sensitivity Analysis: If sensitivity analyses were not conducted to assess the robustness of the findings to different modeling assumptions or potential outliers, this could raise questions about the stability of the results.*

We thank **Reviewer 2** for this comment. Sensitivity analyses were conducted as outlined under statistical analysis in methods (results in supplemental table S4). In sensitivity analyses, models 1-5 were i. stratified by sex and ii. repeated after excluding participants based on immigration status for those who had been in Canada for less than ten years (n=5885). Moreover, as mentioned in our response to **Reviewer 1**, we have now added to our sensitivity analyses, iii. a stratified analysis based on workplace location (working outside the community) to address possible exposure misclassification.

*6. Concerns Regarding Exposure Assessment: The methodology used to gauge individual-level exposures, relying solely on residence addresses, might lead to inaccuracies in exposure classification. The failure to account for variations in participants' locations and their residential histories could compromise the integrity of the exposure evaluation.*

We have addressed this concern in our response to **Reviewer 1**; please see above under point #1.

*7. Inconsistency in Exposure Time Frame: The application of a uniform 5-year window for pollutant exposure across all participants, irrespective of their specific enrollment dates, could introduce an element of bias, potentially skewing the assessment of exposure.*

Thank you. We have addressed this limitation above under point #1 and strived to make it clearer and more transparent in the manuscript.

*8. Unanticipated Results: The findings that NO2 was inversely associated with CWV and that there was no discernible connection with PM2.5 were surprising and at odds with certain existing studies. These outcomes prompt questions about possible unaccounted confounding variables or other hidden factors that might have shaped the results.*

We have addressed this concern in our response to **Reviewer 1**; please see above under point #2.

*9. Limitations in O3 Data Resolution: The 10 km spatial resolution used for O3 data might be insufficient to detect localized fluctuations, which could have an impact on the precision of the conclusions drawn regarding O3.*

We have addressed this concern in our response to **Reviewer 1**; please see above under point #3.

*10. Omission of Intraplaque Hemorrhage Analysis: The study's inability to examine intraplaque hemorrhage, owing to its limited scope, might overlook a crucial aspect in unraveling the complex relationship between air pollution and atherosclerosis. This limitation could be significant in the overall interpretation of the findings.*

We have addressed this concern in our response to **Reviewer 1**; please see above under point #4.

We thank **Reviewer 2** for the detailed comments for improvement of the manuscript.

Of note, we have added 2 new references to the manuscript’s reference list (37 and 38).

We thank the **Editor** and external **Reviewers** for their feedback and hope that our revised manuscript is now deemed acceptable for publication in *PLOS One*.
