# Supplementary material for: Exposure to air pollutants and subclinical carotid atherosclerosis measured by magnetic resonance imaging: A cross-sectional analysis
Source: PLoS One. 2024 Oct 31;19(10):e0309912. doi: 10.1371/journal.pone.0309912 (PMC11527219; doi:10.1371/journal.pone.0309912)
Supplement: Attachment — Submitted filename: Response to Reviewers.docx [file pone.0309912.s007.docx]

13 March, 2024

Muhammad Maaz Arif, M.B.B.S, M.Phil
Academic Editor
*PLOS ONE*

Response to second review: [PONE-D-23-23654R1] ‘Ambient Air Pollution and Subclinical Carotid Atherosclerosis Measured by Magnetic Resonance Imaging: A Prospective Cohort Study’

Dear Dr. Arif,

We are pleased to resubmit our manuscript to *PLOS ONE* following the 2^nd^ review. We have edited the manuscript and provide a point-by-point response to your comments. We feel based on your comments, and those from reviewers, that the manuscript quality has been strengthened and we look forward to the final decision of the revised manuscript.

Please note that the editorial and reviewers’ comments are in blue, while our responses follow in normal font. We have tracked all changes made to the revised manuscript based on the reviewers’ comments in one copy of the manuscript.

Sincerely,

Russell de Souza, Sc.D., R.D.

Associate Professor, Population Genomics Program

Department of Health Research Methods, Evidence, and Impact

1200 Main St. West

MDCL, Room 3210

Hamilton, Ontario, Canada

L8N 3Z5

Phone (905) 525-9140 x. 22109

[desouzrj@mcmaster.ca](mailto:desouzrj@mcmaster.ca)

**ACADEMIC EDITOR Comments:**

Following changes are the top-most priority considering the reviewers comments:
1. Although "air pollution" is cited in the study title, "air pollutants exposure" is explicitly addressed in the findings. Therefore, the study should use more precise language to avoid drawing readers' attention away from the discrepancy between the title and the findings. Suggested Title: Exposure to Air Pollutants and Subclinical Carotid Atherosclerosis Measured by Magnetic Resonance Imaging: A Prospective Cohort Study.

We thank **the Academic Editor** for this suggestion; we have changed the title accordingly.

2. Tables S4 through S8 appear to include the study's most significant findings. Given that these are the most significant findings, why are these tables listed in the supplementary materials? These should be mentioned in the main manuscript.

Table S4 is summarized in Figure 1 in the main manuscript; Table S5 is additional (secondary) sensitivity analysis while tables S6-S8 are all succinctly summarized in Figure 2. Thus, we chose to keep these tables as supplemental to avoid redundancy, while presenting the main messages of these analyses through the figures in the main manuscript. The additional detail is available for detailed review, should a reader wish, but we felt the current presentation to be easier to understand. However, if you feel very strongly, we can move these to the main text.

3. Please take into account the feedback from the reviewers and thoroughly proofread the study for errors and grammatical flaws.

We thank **the Academic Editor** for this comment and have now proofread and revised the article accordingly.

4. The manuscript may be accepted after minor revisions.

We thank **the Academic Editor** for re-reviewing our revised manuscript and for all the valuable insights to strengthen our manuscript.

**Reviewer's Comments:**

The study aimed to investigate the effect of long-term exposure to air pollution on cardiovascular mortality. Therefore, the authors used data from a cohort study of 6,645 individuals recruited from previous Canadian cohort studies. The strength of this study is the well assessed outcome of carotid vessel wall volume (CWV) by MRI, the four-year average duration of air pollution exposure, and the extensive adjustment for confounders including walkability and neighborhood socioeconomic status.

We thank **the** **Reviewer** for echoing the strengths and comprehensive analyses conducted within the Canadian Alliance for Healthy Hearts and Minds (CAHHM).

Interestingly, PM2.5 and NO2 were not or negatively associated with CVW, which requires further investigation.

The reviewers' comments were understandable, and the authors responded to and addressed all comments thoroughly. Furthermore, the feedback has been extensively implemented, including additional sensitivity analyses, resulting in a substantial improvement of the manuscript, which adequately discusses the present limitations. However, there are still a few minor points that need to be addressed:

We thank **the Reviewer** for taking the time to evaluate our revised manuscript and for the favourable response.

\## Minor

-Abstract

for the effect of NO2, a “minus” is missing in the 95%-CI, it must be -7.32

Thank you for noting this error; this is now corrected.

- Methods

More description is needed on the exclusion criterion of "known CVD history". Is this validated or self-reported? And what diseases were included in the CVD history?

We have now added how CVD history was defined as following: (defined as a self-reported history of stroke, coronary artery disease, heart failure, or other heart disease).

o For clarification: add a sentence describing whether residential addresses were only available on a postcode grid. If so, then PM2.5 available on a 1km*1km grid was probably averaged over the postcode area? This is not clearly described in the methods section.

The following paragraph has now been added to the methods section:

“For most residential addresses, postal code areas were considerably smaller than 1x1 km so that the assigned PM_2.5_ concentration matches the 1x1 km grid square that the postal code is found within. Specifically, assigning PM_2.5_ to postal codes was performed using the single linkage approach where the PM_2.5_ grid square selected was the one closest to the x, y coordinate within a postal code polygon that best represents where the majority of the population lived.”

o As an additional analysis, the ratio of wall volume to total vessel volume (Normalized wall index) should be used instead of using maximum wall volume [e.g. PMID: 33183741]. This would account for individual variation in overall vessel size.

We thank **the Reviewer** for this suggestion and reference. However, we prefer to use CWV as our outcome because we have previously validated it in CAHHM where we’ve published CWV ranges related to cardiovascular risk; which is useful clinically [Anand SS, Tu JV, Desai D, et al. Cardiovascular risk scoring and magnetic resonance imaging detected subclinical cerebrovascular disease. *Eur Heart J - Cardiovasc Imaging*. 2020;21(6):692-700]. Of note, in the sensitivity analysis stratified by sex to account for sex variation, all results were consistent between men and women.

We have now added this sentence to our Discussion (this also addresses the Reviewer’s comment below on clinically relevant cut-off values for CWV):

“We have previously shown in CAHHM how simple cardiovascular risk scores were significantly associated with CWV, where mean (SD) CWV for low, medium, and high INTERHEART risk score categories were 881.5 (163.1), 915.4 (166.6), and 940.9 (172.9) mm^3^, respectively.”

If the **Academic Editor** still would like to have this additional analysis, we can conduct it.

-Results:
o Information in the text do not match the information in the table, e.g., in the Results - participant characteristics section it says "54.8% of participants were women", but in Table 1 it says 56%. Same section: "92% of cohort postcodes were in urban areas", but in Table 3 it is 96.2%. It seems that information in the text was mixed up with the “British Columbia” column. Authors should double-check tables and text to correct any discrepancies.

We thank **the Reviewer** for noting this error. This change has been made for % women. As for urbanicity, we wrote that: “For all the regions, ***over*** 92% of the cohort’s postal codes were in urban areas.” i.e. that the minimum % for ***any of*** the provinces was 92%.

-Discussion
Any clinically relevant cut-off values for CWV? Is the mean value high (900 mm³)? Could be more addressed in the discussion and may help to contextualize the results and health of study participants to previous studies.

We thank **the Reviewer** for this valuable suggestion which also reinforces our choice of using CWV as our outcome measure. As noted above we have added the following sentence to the discussion: “We have previously shown in CAHHM that simple cardiovascular risk scores were significantly associated with CWV, where mean (SD) CWV for low, medium, and high INTERHEART risk score categories were 881.5 (163.1), 915.4 (166.6), and 940.9 (172.9) mm^3^, respectively. Therefore, the overall mean in the current analysis set (900 mm^3^) corresponds to someone with a low-moderate IHRS.”

I agree with Reviewer 2 that the study was rather cross-sectional in nature than longitudinal. The authors argue “Exposure to air pollution values represented a time frame prior to knowledge of the outcome for each participant, i.e., the air pollution data collected for the 5-year period prior to the MRI. We are therefore comfortable describing this as a prospective association, despite the lack of longitudinal follow-up.” However, since no information exists on the CVW thickness before the exposure window, one cannot argue that the thickness was not present at this exposure window or even before. Therefore, I suggest revising this sentence and to name it a cross-sectional association.

We acknowledge that the study design is not straight-forward because CAHHM itself is a prospective cohort study and the air pollution exposure measures are in the years prior to the outcome measurement but recognize that these air pollution estimates are rather stable over time in the years to follow as well (as noted in the discussion). Thus, to adopt a more conservative approach and because of the lack of longitudinal follow-up, we have now changed the study design to cross-sectional analysis in the title and abstract and have removed this sentence from the discussion.

Optional: instead of A, B, C, in the plots, naming the air pollutants would improve the readability of the plots.

We thank **the Reviewer** for this optional comment. We prefer to leave as A, B, C, to avoid confusion in case of naming of the air pollutants in Figure 2 where we are testing the interactions for each pollutant within the other 2 pollutant tertiles.

We thank the **Academic Editor** and external **Reviewer** for their feedback and hope that our revised manuscript is now deemed acceptable for publication in *PLOS One*.
